# Luminol–hydrogen peroxide–horseradish peroxidase chemiluminescence intensification by kosmotrope ammonium sulfate

**DOI:** 10.1007/s44211-022-00069-8

**Published:** 2022-02-15

**Authors:** Hajime Karatani

**Affiliations:** Kyoto Luminous Science Laboratory, Keihanna Plaza, Laboratory Wing, 1-7 Hikaridai, Seika-cho, Soraku, Kyoto 619-0237 Japan

**Keywords:** Luminol, chemiluminescence, kosmotrope, ammonium sulfate, hydrogen peroxide, horseradish peroxidase

## Abstract

The kosmotropic effect induced by ammonium sulfate (AS) at concentrations greater than approximately 2.8 M allows the marked intensification of chemiluminescence (CL) arising from a conventional luminol–hydrogen peroxide (H_2_O_2_)–horseradish peroxidase (HRP) reaction. Because of the kosmotropic effect, CL is intensified by at least three orders of magnitude than that from the conventional HRP-catalyzed luminol reaction with no AS; the linear relationship between the CL intensity and the HRP concentration is established over the range of 0.3 pM to several tens of pM. The novel CL intensification effect on the HRP-catalyzed luminol CL can be stably and reproducibly induced.

**Graphical abstract:**

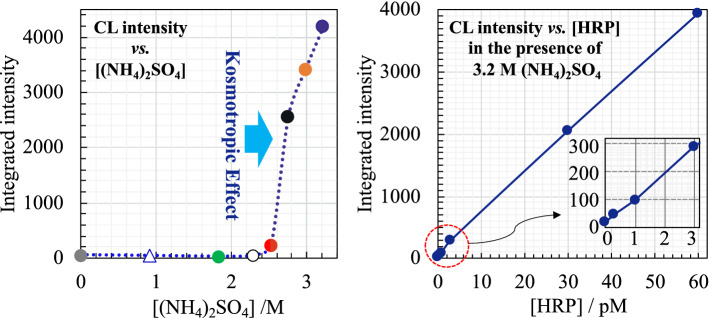

**Supplementary Information:**

The online version contains supplementary material available at 10.1007/s44211-022-00069-8.

## Introduction

Chemiluminescence (CL), as a result of the chemiexcitation derived from a series of chemical reactions, is an effective tool in analytical chemistry [1]. One of the applications of CL that has high practicability is the CL derived from a luminol–H_2_O_2_–horseradish peroxidase (HRP) reaction, wherein HRP is frequently used as an antibody marker. For example, in the enzyme-linked immunosorbent assay (ELISA), HRP is used as a marker enzyme for the secondary antibodies and the highly sensitive detection of HRP-linked antibodies is attained based on the luminol CL [2].

The reaction mechanism for the CL arising from the luminol–H_2_O_2_–HRP reaction is described in Scheme S1 (Supporting Information). The key steps in enzymatic turnover are the formation of luminol radical (LH·) via the successive oxidation of luminol monoanion (LH^–^) by the HRP intermediates at around pH8.5 (Fig. S1 (A) and (B) (Supporting Information)), described as Eqs. (S2), (S3), and (S4) in Scheme S1 [3, 4]. Usually, the CL arising from a conventional luminol–H_2_O_2_–HRP reaction is not so strong, partly because the HRP-catalyzed luminol reaction requires a pH of < 9 to prevent a decline in the enzymatic activity. Such a pH is relatively low to produce the strong CL in the luminol reaction. Hence, the intensification of the HRP-catalyzed luminol CL is crucial for expanding its application in highly sensitive assays, such as the ELISA and CL enzyme immunoassay (CLEIA).

To date, many efforts have been devoted to intensifying the luminol–H_2_O_2_–HRP CL. Kricka et al*.* achieved excellent results with their method, in which *p*-substituted phenol derivatives, serving as enhancer agents, were used to accelerate the formation of LH·. In brief, the formation of LH· is accelerated by two to three orders of magnitude via reaction steps, described as Eqs. (S8), (S9), and (S10) in Scheme S1 (Supporting Information) than that of the conventional HRP-catalyzed formation of LH· [5–10]. Such accelerated formation of LH· is considered crucial for enhancing CL. Luminol–H_2_O_2_–HRP CL with an enhancer agent has been widely utilized as a highly sensitive detection signal for ELISA and CLEIA [1, 2, 11]. Recently, the performances of various enhancers have been reported in detail [12].

With respect to the intensification of the luminol–H_2_O_2_–HRP CL, it is worth noting that a peroxidase from *Anthromyces ramosus* (referred to as ARP) catalyzes the luminol reaction to produce considerably stronger CL than HRP does even in the absence of an enhancer agent [13, 14]. The *Anthromyces ramosus* peroxidase was also applied to CLEIA and was shown to detect 5 × 10^−13^ M (0.5 pM) ARP [14]. A more recent report has shown that the peroxidase isolated from sweet potato (*Ipomoea batatas*) (referred to as SPP) also acts as a very efficient catalyst for the luminol CL to allow for the detection of 1 × 10^−14^ M (10 fM) SPP [15].

Notably, the luminol reaction steps, resulting in the production of photons, are generally susceptible to changes in the microscopic environment of the reaction domain. To date, there have been many reports on the analytical applications of various microenvironmental effects on the luminol CL including our previous result [16]. Focusing on the microscopic hydrophobicity, it is important to note that the luminol CL is enhanced in micelles [17, 18]. It should also be noted that the HRP-catalyzed luminol CL with an enhancer agent is favorably triggered in liposomes [19].

From the viewpoint of the microscopic hydrophobicity, it is highly expected that the kosmotropic effect exerts an effect on the luminol CL reaction. Several studies have shown that the ions dissociated from kosmotropic salts, such as ammonium sulfate (referred to as AS), interact with the water molecules forming the hydration shell of a protein molecule [20]. In particular, SO_4_^2−^ exhibits distinct kosmotropic properties to remove water molecules from the protein surface [21]. Such interactions would occur in various chemical species. For example, water molecules bound to the hydroxy groups of cellulose are removed in the presence of high concentrations of NH_4_^+^ and SO_4_^2−^, resulting in the increase in the hydrophobic interaction between protein molecules and the hydroxy groups [22]. Based on these reports, it is expected that the hydrogen-bonded water molecules of the luminol reaction species are also removed via the interactions with NH_4_^+^ and SO_4_^2−^, existing in high concentrations. This may result in an increase in the hydrophobic microenvironment around molecules, ions, and possibly radicals involved in the luminol reaction, resulting in the intensification of CL.

In this study, it was shown that the CL from an HRP-catalyzed luminol reaction was markedly intensified in the presence of high concentrations of AS by at least three orders of magnitude than that from the conventional luminol–H_2_O_2_–HRP reaction without using the so-called enhancer agent. The novel CL intensifying system established in this study was studied from the viewpoint of the kosmotropic effect with the aim of analytical applications.

## Experimental

### *Chemicals*

3-Aminophtalhydrazide (luminol) was used for biochemistry (Fujifilm Wako Pure Chemical Co., Osaka, Japan). AS was for enzyme refining (Fujifilm Wako). Hydrogen peroxide solution for the atomic absorption spectrochemical analysis was also obtained from Fujifilm Wako. Horseradish peroxidase was used for biochemistry (Fujifilm Wako). Tris(hydroxymethyl)aminomethane (referred to as Tris) was used for molecular biology (Fujifilm Wako). Ethylenediamine-*N*,*N*,*N′*,*N′*-tetraacetic acid (EDTA), disodium salt, dihydrate was purchased from DOJINDO (Kumamoto, Japan). 3-Aminophtalic acid (3-AP), hydrochloride dihydrate was purchased from FluoroChem Ltd. (Hadfield, Derbyshire, UK). Sodium hydroxide (NaOH) used was a Fluka’s MicroSelect. *Ortho*-phenylenediamine (OPD) was purchased from Fujifilm Wako. Other reagents were of the highest commercial grade and were used as received. Solutions were all prepared using distilled water (Fujifilm Wako).

### *Reagent solutions*

The HRP stock solution was first prepared in a phosphate (0.05 M) buffer solution (pH7.2); the concentration of the stock solution was typically set between 3 × 10^−4^ and 5 × 10^−4^ M. The concentration was determined based on the molar absorption coefficient of HRP at 403 nm (1.02 × 10^5^ M^−1^ cm^−1^) [14]. The HRP stock solution was divided into small amounts (30 μL each) and stored at − 20 °C in a frozen state. When used, the HRP stock solution was thawed on ice and then diluted with the Tris (0.10 M) buffer solution (pH8.5) containing 3.5 M AS. The final pH of the Tris buffer solution containing AS was adjusted with 6 M HCl or 6 M NaOH to approximately 8.5. Luminol was first dissolved in a 0.75 M NaOH solution to a concentration of 30 mM and subdivided into small volume (10 cm^3^ each) for storage at 4 °C in the dark. The luminol stock solution was mixed with pH8.5 Tris (0.10 M) containing various concentrations of AS at a volume ratio of 1:5. In the AS-free system, luminol was first dissolved in a small amount of the 0.75 M NaOH solution and then diluted with a Tris (0.10 M) buffer solution (pH8.5) to achieve a concentration of 5.0 mM. NaOH concentration in this mixture was approximately 190 mM.

H_2_O_2_, whose concentration was determined at 12.2 M using a potassium permanganate titration, was diluted with the AS solution containing 1000 ppm EDTA and then subdivided into 10 cm^3^ each for storage at 4 °C in the dark until use. H_2_O_2_ concentration was 100 mM at this point. In the AS-free mixture, 6 M HCl (25 μL) was added to H_2_O_2_–EDTA (20 mL) to adjust the pH of the final reaction mixture to approximately 8.5.

For CL measurement, the reagent solutions were mixed as follows: luminol solution containing AS and H_2_O_2_ solution containing AS were gently premixed at a volume ratio of 1:1. Next, 1.0 mL of this mixture was rapidly added to 1.0 μL of various concentrations of HRP diluted with the Tris (0.10 M) buffer solution (pH8.5) containing 3.5 M AS, placed in a plastic cuvette in advance, and the CL spectrum was recorded as a function of time. A mixture of AS-free luminol solution and AS-free H_2_O_2_ solution was used as the control reaction. The time required to start the spectrum recording was fixed at 10 s (0.166 min), and the spectrum recording was carried out at a time interval of either 0.166 min or 1 min. The wavelength scan rate was fixed at 50 nm s^−1^. The width of the monochromator slit for the CL measurement was also fixed at 20 nm. The area under each spectrum (350–550 nm) was regarded as the integrated intensity. All measurements were performed at room temperature (25 ± 1 °C).

### *Apparatus*

CL and fluorescence spectra were measured using a spectrofluorophotometer (RF5300PC, Shimadzu, Kyoto, Japan). During the CL measurement, the excitation lamp was turned off. Absorption spectra were recorded using a UV–Vis spectrometer (V630, JASCO, Hachioji, Japan). pH was monitored using a pH/COND Meter D-24 and a model 6366-10D pH electrode (Horiba, Kyoto, Japan).

### *Measurements of fluorescence and CL spectra*

To record the fluorescence emission spectra, excitation light sources were fixed at 340 nm for luminol and at 300 nm for 3-AP. In contrast, a fluorescence at 450 nm was used to monitor the excitation spectra of both luminol and 3-AP. The monochromator slit widths for excitation and emission were both fixed at 3 nm. The fluorescence spectra of the final CL reaction mixtures were also recorded. In this case, the initial concentrations of luminol, H_2_O_2_, and HRP were adjusted to10 μM, 100 mM, and 3 μM, respectively, to complete the reaction overnight at 20 °C. The background fluorescence including the Raman scattering light was subtracted from the fluorescence spectra. Fluorescence spectra obtained were subjected to the 21-point smoothing with Igor Pro 6.3 (WaveMetrics, OR, USA).

### *Evaluation of effect of AS on HRP activity*

The effect of AS on the HRP activity was evaluated using the OPD–H_2_O_2_–HRP conjugate [23]. Details of the experimental procedure are described in “HRP activity against (NH_4_)_2_SO_4_ concentration” (Supporting Information).

## Results and Discussion

### *Effect of high concentration AS on CL*

**Fig. 1:**
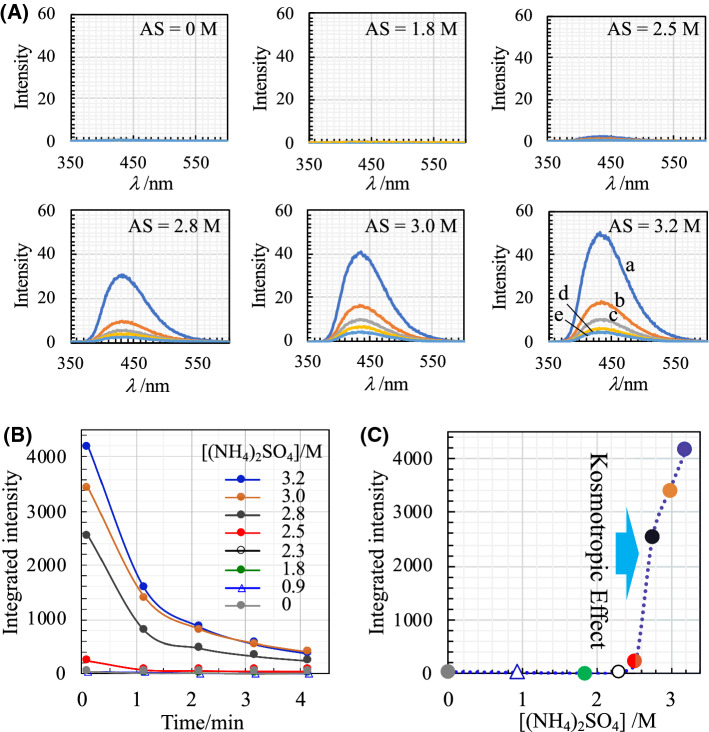
Effect of ammonium sulfate (AS) on luminol–H_2_O_2_–HRP CL. **A** Changes in CL spectra as a function of time. Spectral measurements are started at 10 s (0.166 min (**a**)) after the initiation of the reaction and then are recorded at a time interval of 1 min: **b** 1.166; **c** 2.166, **d** 3.166, and **e** 4.166 min. Concentration of AS in the reaction mixture is designated on each panel. Initial concentrations of HRP, H_2_O_2_, luminol, and EDTA in the reaction mixture were 1.0 × 10^−10^ M, 50.0 mM, 2.50 mM, and 500ppm, respectively. **B** Plots of integrated intensities of CL spectra in (**A**) against time. **C** Replots of integrated intensities of the 1st CL spectra against AS concentration. Light blue rectangle indicates the concentration region at which the distinct effect of AS on CL intensification is induced

The CL outputs observed in the presence of various concentrations of AS are representatively shown in Fig. 1. It is evident that the CL arising from the luminol–H_2_O_2_–HRP reaction is markedly intensified in the presence of high concentrations of AS (Fig. 1A). A striking event is that the CL is abruptly intensified when the concentration of AS exceeded approximately 2.8 M (Fig. 1B). This was clearly demonstrated by replotting the integrated intensity against the AS concentration of the reaction mixture (Fig. 1C).

Such an abrupt jump in CL intensity suggests that the threshold AS concentration value, at which the CL intensification is triggered, exists. Thus, CL intensification induced by the effect of high concentrations of AS is a type of non-linear phenomenon. At AS concentrations greater than the threshold value, the effect on CL intensification was stably and reproducibly induced. Because the solubility of AS is considerably higher (76.4 g/100 g H_2_O at 25 °C) [24] than that of other kosmotropic salts, such as (NH_4_)_2_CO_3_ and Na_2_SO_4_, increasing the concentration up to approximately 4 M is possible. However, to prevent crystal deposition during long-term storage at lower temperatures, the AS concentration in the stock solutions was set at 3.5 M in this study.

### *Hofmeister effects on HRP*

AS is a representative kosmotrope and exhibits strong Hofmeister effects, resulting in the salting-out of proteins [25–27]. In this context, it is important to ensure that the salting-out of HRP does not occur in the CL reaction solution. For this purpose, the absorption spectra of various concentrations of HRP in the presence and absence of 3.5 M AS were measured, and the results are shown in Fig. S2 (Supporting Information). As shown, the background absorption increased in the presence of 3.5 M AS when the HRP concentration was at the μM level. The HRP solution became faintly cloudy over time. However, the HRP solution returned to the original transparent solution on dilution with the Tris (0.10 M) buffer solution (pH8.5) containing 3.5 M AS. When the HRP concentrations were lower than sub-μM level, no hike was observed in the background absorption. Thus, it can be concluded that a high concentration of AS does not promote the salting-out of HRP in the reaction solution whose HRP concentration was lower than sub-μM level. As the concentrations of HRP in ELISA and CLEIA are usually much lower than the sub-μM level, the salting-out of HRP can be prevented.

### *Removal of background CL*

Removing background CL is essential for analytical applications. Background CL is frequently brought about by contaminants in the reagents. In the present system, trace amounts of metal salts, possibly in high concentrations of AS, may be responsible for the background CL. Indeed, CL was faintly observed in the HRP-free reaction containing high concentrations of AS. Therefore, to remove this background CL, the effectiveness of EDTA was examined. As a means of use, EDTA was dissolved in an H_2_O_2_ solution containing 3.5 M AS. CL outputs in the presence and absence of EDTA are representatively shown in Fig. S3 (Supporting Information).

As stated above, weak CL occurred in the HRP-free reaction with high concentrations of AS in the absence of EDTA (Fig. S3, left dotted panel, bottom). This corresponds to the background CL. As a result, such background CL overlapped the signal CL (Fig. S3, right dotted panel, bottom). It was found that the background CL was almost completely removed upon EDTA addition (Fig. S3, left dotted panel: EDTA in the reaction mixture, 500ppm). It is important to note that EDTA does not interfere with the HRP-catalyzed luminol CL. It is also anticipated that EDTA acts as a H_2_O_2_ stabilizer because of its long storage time. Considering the roles of EDTA as a H_2_O_2_ stabilizer and a background remover, EDTA was added to the H_2_O_2_ stock solution containing 3.5 M AS to be at 1000ppm.

### *Concentrations of H*_*2*_*O*_*2*_*, luminol, and Tris base*

From a practical viewpoint, the concentrations of H_2_O_2_, luminol, and Tris base were examined; the results are shown in Fig. S4 (Supporting Information). Regarding the concentration of H_2_O_2_, there were no considerable differences in the CL outputs between 50 and 300 mM in the H_2_O_2_ solution containing EDTA (1000ppm) and AS (3.5 M), and 100 mM of H_2_O_2_ was employed in this study (Fig. S4 (A)). In the case of luminol, too, there were also no considerable differences in the CL output between 3.4 and 6.8 mM in the luminol solution containing 3.2 M AS (Fig. S4B) and 5 mM luminol was used. The concentration of NaOH to prepare a luminol stock solution was found to be suitable at 0.75 M for to make the pH of the final reaction mixture to be approximately 8.5. Regarding Tris base, a significant difference in the CL output was not present among the three concentrations examined (Fig. S4C) and 0.10 M of Tris base was used. It should be stressed that no CL emission was observed in any of the HRP-free reactions in the presence of EDTA. Regarding the mixing order of the reagent solution, it was confirmed that the addition of a premixture of equal volumes of H_2_O_2_ solution and luminol solution to a small amount of the HRP solution produced stronger CL rather than adding them to HRP individually.

In terms of the stability of reagent stock solutions, H_2_O_2_ stock solution containing 1000ppm EDTA and 3.5 M AS, Tris (0.10 M) buffer solution (pH8.5) containing 3.5 M AS, the luminol stock solution in 0.75 M NaOH, and the HRP stock solution in phosphate (0.05 M) buffer solution (pH7.2) were all stable for at least a couple of months. If the luminol stock solution was mixed with a Tris (0.10 M) buffer solution (pH8.5) containing 3.5 M AS, it is preferable to use the mixture within a day. However, by storing it at 4 °C, its performance can be retained for a few days.

### *Effect of AS on HRP activity*

It is interesting to determine whether AS influences on the HRP activity. For this purpose, it is desirable to track the formation of LH·, described as Eqs. (S3) and (S4) in Scheme S1 (Supporting Information) as functions of time. However, the measurement of LH· is not easy, because it is swiftly subjected to the following reaction, described as Eq. (S5) in Scheme S1 (Supporting Information). On a trial basis, the HRP reaction with OPD instead of luminol was employed. This system is relatively sensitive and has been used to determine relatively low concentrations of HRP [23].

Figure S5 (Supporting Information) shows the plots of the relative HRP activity (= Δ*A*_with AS_/Δ*A*_without AS_; Δ*A*, the increase in integrated absorbance at 417 nm for 1 min after the initiation of the reaction) against AS concentration. As shown, the enzymatic activity of HRP was gradually enhanced with an increase in the concentration of AS and reached a maximum at around 1.8 M, in contrast to the observation that the CL intensity began to increase when the AS concentration became greater than approximately 2.8 M (Fig. 1C). However, the increase in the HRP activity caused by 3.2 M AS is no more than approximately five times that in the AS-free system. This observation may be explained by the idea that NH_3_(aq) partially produced at pH8.5 in the Tris buffer solution containing 3.2 M AS (Fig. S1D (Supporting Information)) partly enters the HRP binding site. This idea is based on the report on the crystallographic study describing that the properties of the heme iron of *Arthromyces ramosus* peroxidase are affected by the high concentrations of AS [28].

The observed increase in the HRP activity in the presence of high concentrations of AS would contribute to increase the CL intensity. However, this effect would not be the most crucial factor in intensifying CL.

### *Microscopic environment surrounding reactants*

Generally, the CL intensity at any time *t* (*I*_*t*_) can be expressed as the product of the CL quantum yield (*Φ*_CL_) and the reaction rate for the key CL reagent (*dC*(*t*)*/dt*) [29], that is, *I*_*t*_ =  *Φ*_CL_·(*dC*(*t*)*/dt*). *Φ*_CL_ is the product of the total reaction yield (*Φ*_*r*_), the efficiency of raising the product to its excited state (*Φ*_es_), and its fluorescence quantum yield (*Φ*_FL_) [30, 31], that is*, Φ*_CL_ = *Φ*_*r*_·*Φ*_es_·*Φ*_FL_.

As mentioned above, it is assumed that a high concentration of sulfate ion dissociated from AS gives rise to hydrophobicity in the reaction domain of HRP. In relation to this assumption, it is interesting to see whether high concentrations of AS cause changes in the microenvironment surrounding the luminol reaction species. For this purpose, fluorescence and CL spectral characterizations were carried out in the presence and absence of AS.

**Fig. 2:**
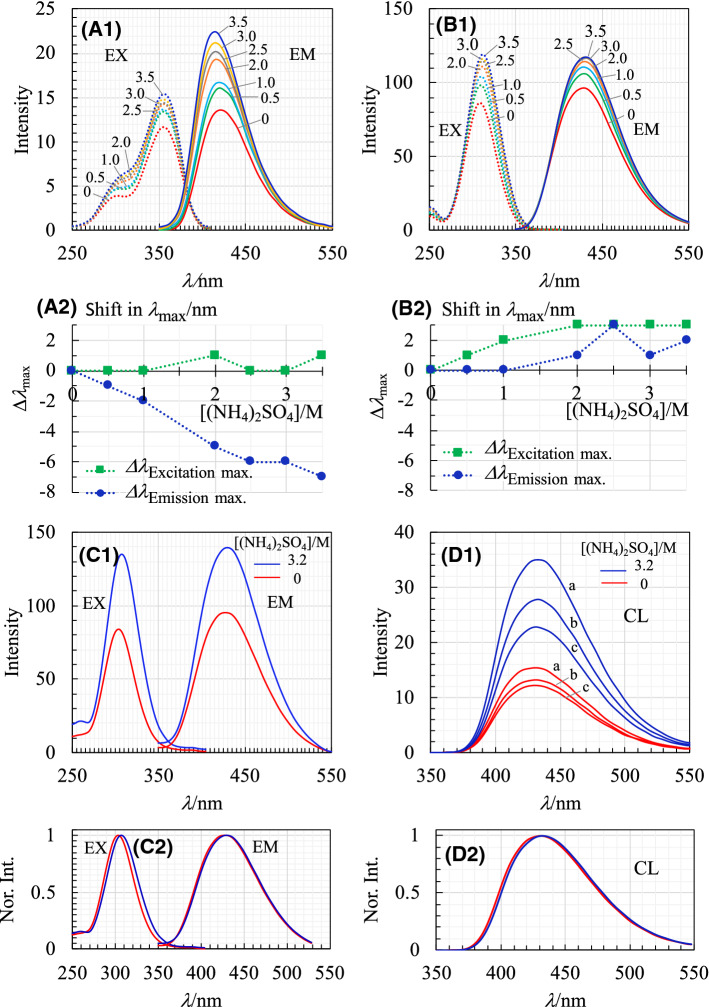
Fluorescence emission (abbreviated as EM) and excitation (EX) spectra of luminol (**A-1**), 3-AP (**B-1**), and the final CL reaction mixture (**C-1**); and CL emission spectra (**D-1**). For **A-1** and **B-1**, concentrations of luminol and 3-AP are both 10.0 μM in pH8.5 Tris (0.10 M)-HCl buffer solution containing various concentrations of AS (/M), which are designated on each graph. **A-2** and **B-2**, plots of the shift in *λ*_max_ of EX and EM spectra for luminol and 3-AP against AS concentration. Spectra in (**C-2**) are normalized to each peak intensity in (**C-1**). CL spectral scan is started at 10 s after the initiation of the CL reaction, and then recorded at a 10-s interval. CL spectra in (**D-1**) are obtained during 2nd (**a**), 3rd (**b**), and 4th (**c**) scans in the presence of 3.2 M AS (blue) and in its absence (red). All CL spectra are subjected to 21-point smoothing. Initial concentrations of luminol and H_2_O_2_ in the reaction solution are 2.5 mM and 50.0 mM. Concentrations of HRP are 96 nM in the reaction free of AS and 60 pM in the reaction with 3.2 M AS. CL spectra in (**D-2**) are also normalized to each peak intensity in (**D-1**)

As shown in Fig. 2A-1 and A-2, the fluorescence emission peak of luminol shifted toward shorter wavelengths in the presence of AS (approximately 7 nm at the maximum), although there was no substantial shift in the excitation spectra. Based on the knowledge that a blue shift is usually caused by the increase in hydrophobicity [32], the observed blue shift can be explained by the idea that the water molecules forming the hydration shell of LH^−^, a major luminol species at pH8.5 (Fig. S1B (Supporting Information)), were eliminated to some degree in the presence of high concentrations of AS. If this is the case, then LH^−^ may more easily access the reaction domain of HRP whose hydrophobicity is likewise increased in the presence of high concentrations of AS.

Both the fluorescence excitation and emission spectra of 3-AP, possibly existing in the dianion form as deduced from p*K*_a1_ and p*K*_a2_ of phthalate (2.94 and 5.43), [33] faintly moved toward longer wavelengths (Fig. 2B-1 and B-2). Considering that these changes are very small, it seems that the interaction between the hydration water molecules of 3-AP and the high concentration of SO_4_^2−^ as well as NH_4_^+^ is relatively weak.

It is also important to note not only that the spectral distribution of CL in the presence of 3.2 M AS is in rough agreement with that in its absence (Fig. 2D-1 and D-2) but also that CL spectral distributions are in rough agreement with those of the fluorescence emission of the final CL reaction mixture as well as 3-AP (Fig. 2B-1, C-1, and C-2). From these observations, it is considered that CL emission occurs in the bulk solution but not in the reaction domain of HRP.

Focusing on the hydration shell, H_2_O_2_ is also expected to release the hydration water molecules, possibly leading to the facilitation of the reaction between H_2_O_2_ and luminol diazaquinone (Eq. (S6) in Scheme S1 (Supporting Information)) as well as between HRP and H_2_O_2_ (Eq. (S2) in Scheme S1). Furthermore, taking the molar fractions of NH_4_^+^ and NH_3_ as a function of pH (Fig. S1 (D) (Supporting Information)) into consideration, it is conceivable that NH_3_(aq) is partially produced via the Brønsted–Lowry acid–base reaction between NH_4_^+^ and H_2_O in the reaction mixture (pH 8.5) containing 3.2 M AS. The resultant NH_3_(aq) will cause a partial dissociation of the weak acid H_2_O_2_ into HO_2_^−^, whose reaction with luminol diazaquinone is more efficient than that of the fully protonated H_2_O_2_. Thus, the expected interactions between the hydration water molecules of the reactants and high concentrations of AS are considered to contribute to increasing the product *Φ*_*r*_·*Φ*_es_ as well as the total reaction rate resulting in CL intensification.

The fluorescence emission intensities of both luminol and 3-AP increased in the presence of high concentrations of AS (Fig. 2A–C). Such an increase would also be a contributing factor in intensifying CL. However, this contribution may partly be attributed to the change in the viscosity of the solution in the presence of high concentrations of AS.

To clearly elucidate the effect of kosmotrope AS on the luminol–H_2_O_2_–HRP reaction, detailed studies on the reaction kinetics and quantum yields are required.

### *CL output versus picomolar HRP*

**Fig. 3:**
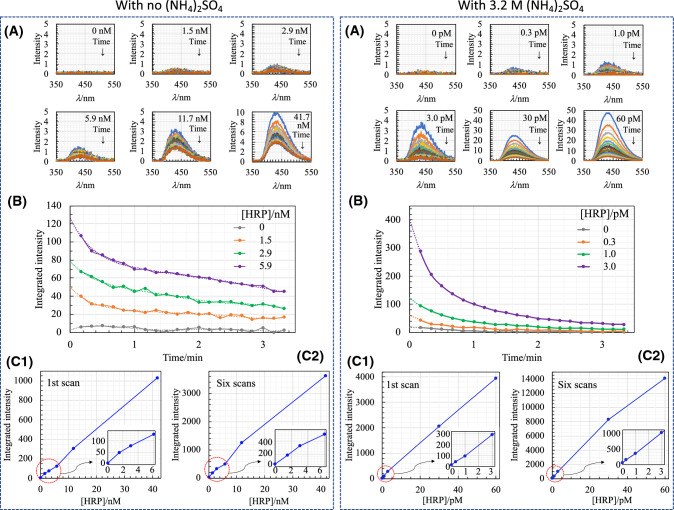
Detection of HRP using the conventional system (left dotted panel) and using the CL system with 3.2 M AS (right dotted panel). **A** Changes in CL spectra as a function of time after the initiation of the reaction; CL spectra are obtained in a manner similar to that in Fig. 1 except for a 10 s interval. Ordinate scale is magnified or reduced according to the CL intensity. **B** Time-courses of CL outputs with low concentrations of HRP in the absence and presence of 3.2 M AS. All time-courses are extrapolated (dotted line) to the time *t* = 0, based on the curve fitting using 6th-degree polynomial function. **C** Plots of CL intensities against the HRP concentration in the reaction mixture; **C-1**, the area of the first CL spectrum; and **C-2**, the area of the six spectra (from 1st to 6th)

Figure 3 shows the CL outputs in the presence of 3.2 M AS and in its absence. It is shown not only that sub-pM HRP is detected on the basis of the proposed CL, but that the linear relationship between the CL output and the HRP concentration is established in the range of 0.3–60 pM. By comparing the CL outputs against the HRP concentration obtained in the presence of 3.2 M AS with those obtained in its absence, it can be concluded that the sensitivity of the luminol–H_2_O_2_–HRP system with 3.2 M AS is at least three orders of magnitude greater than that of the conventional system with no AS. The reproducibility of the measurement, especially at sub-pM and pM levels of HRP, was evaluated, and the results obtained are shown in Fig. S6 (Supporting Information). Judging from the observation that the coefficient of variation (CV = standard deviation/average intensity at each HRP concentration, *n* = 5) is almost smaller than 0.1, the reproducibility of the measurement at sub-pM and pM levels can be regarded as favorable.

Regarding the CL intensity time-course, the time required for the peak intensity to decay to one-half was slightly shorter than 1 min in the presence of 3.2 M AS, as shown in Figs. 1, 3, Fig. S4 (Supporting Information), and Fig. S6 (Supporting Information). This CL intensity decay was several tens of times faster than that observed in the HRP-catalyzed luminol reaction with an enhancer agent [19]. Such a feature of the CL decay observed in this study is somewhat similar to that frequently observed in the metal-catalyzed luminol reaction in the basic aqueous solution [34]. To prolong the CL emission, it would be necessary to improve the reaction conditions from the kinetic viewpoint. Regarding this, there may be a hint in Fig. S4A-1 (Supporting Information), showing that reducing the H_2_O_2_ concentration retards the CL decay, although the intensity in the initial stage slightly decreases.

However, it should be stressed that the novel CL enhancing system established in this study enables the detection of sub-pM HRP without using the so-called enhancer agents, such as *p*-iodophenol [1, 2, 6–12, 19]. At present, the detection of the fM level of HRP has not yet been achieved. To realize this, further improvements in the reaction conditions, particularly to attain long-lasting CL emission, would be necessary. Moreover, it may be of interest to use the proposed CL system in combination with highly active peroxidases, such as ARP [13, 14] and SPP [15].

## Conclusions

In this study, it was shown that the CL arising from the conventional luminol–H_2_O_2_–HRP reaction is markedly intensified in the presence of high concentrations of AS. Such a novel CL intensification is concluded to be the result of the kosmotropic effect induced by high concentrations of AS. It is also important to note that kosmotrope AS generally stabilizes the structure of proteins [26, 35]. This would also contribute to the stable induction of the CL intensification effect on the HRP-catalyzed luminol reaction.

## Supplementary Information

Below is the link to the electronic supplementary material.

Supplementary file1 (PDF 1522 KB)

## Declarations

### Supporting information

Scheme S1, the additional experimental procedure “HRP activity against AS concentration”, and Figs. S1–S6 are available in Supporting Information. This material is available free of charge on the Web at http://www.jsac.or.jp/analsci/.
