# Supplementary material for: Luminol–hydrogen peroxide–horseradish peroxidase chemiluminescence intensification by kosmotrope ammonium sulfate
Source: Anal Sci. 2022 Feb 15;38(3):613–21. doi: 10.1007/s44211-022-00069-8 (PMC8971166; doi:10.1007/s44211-022-00069-8)
Supplement: Supplementary file 1 — Supplementary file1 (PDF 1522 KB) [file 44211_2022_69_MOESM1_ESM.pdf]

Supporting Information

Luminol-Hydrogen Peroxide-Horseradish Peroxidase Chemiluminescence Intensification by Kosmotrope Ammonium Sulfate

Hajime KARATANI

Kyoto Luminous Science Laboratory, Keihanna Plaza, Laboratory Wing, 1-7 Hikaridai, Seika-cho, Soraku, Kyoto 619-0237, Japan

E-mail: karatani@luminous-science.jp

Table of Contents

Scheme S1

HRP activity against $(NH_4)_2SO_4$ concentration

Fig. S1

Fig. S2

Fig. S3

Fig. S4

Fig. S5

Fig. S6

Dissociation of luminol

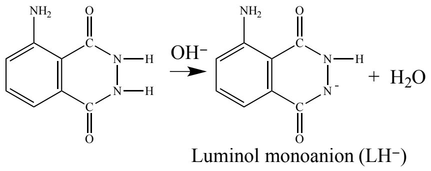

(S1)

First step of the reaction of HRP with H_2O_2

HRP (+3) + H_2O_2 ⟶ Compound I (+5) + H_2O (S2)

Reaction of LH⁻ with Compound I

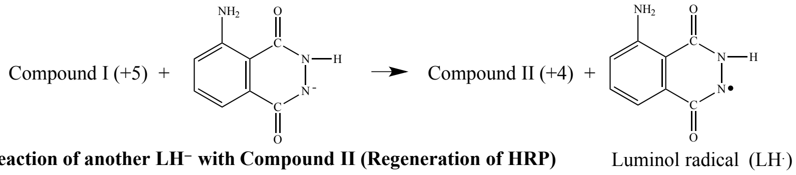

(S3)

Reaction of another LH⁻ with Compound II (Regeneration of HRP)

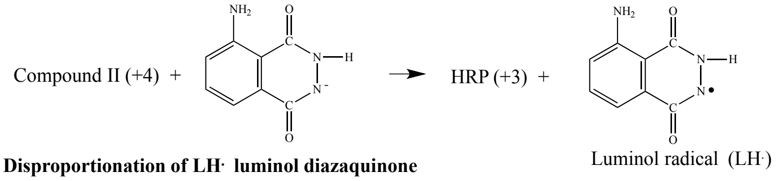

(S4)

Disproportionation of LH· luminol diazaquinone

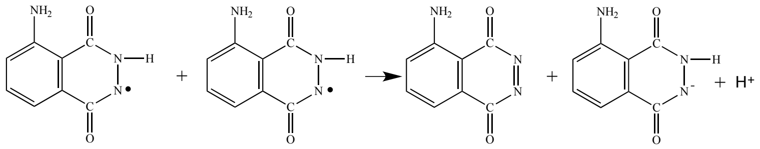

(S5)

Nucleophilic attack of HOOH (or HOO⁻), followed by the formation of luminol dixetane product

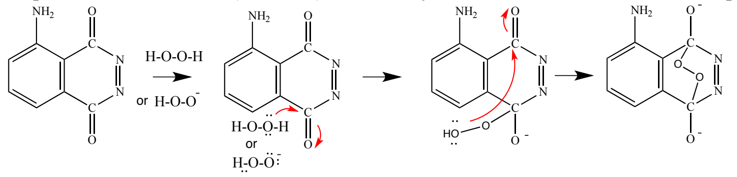

(S6)

Cleavage of dioxetane, followed by the formation of excited 3-AP; and then light emission

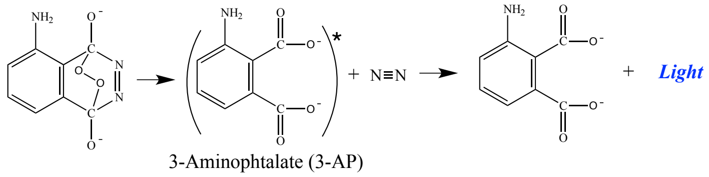

(S7)

In the presence of enhancer agent (SH); *e.g.*, 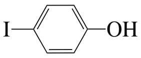

Compound I (+5) + SH ⟶ Compound II (+4) + S· (S8)

Compound II (+4) + SH ⟶ HRP (+3) + S· (S9)

2S· + 2LH⁻ ⟶ 2LH· + 2SH (S10)

Scheme S1 Reaction mechanism for luminol - H_2O_2 -HRP reaction in the absence and presence of an enhancer agent. Explanation for the reaction is described on the following page.

Considering that pK_{a1} of H_2O_2 is 11.62 (Fig. S1 (A) (Supporting Information)),[1] it exists as a fully protonated form in a weakly basic solution. In contrast, as pK_{a1} of luminol is 6.35 (Fig. S1 (B) (Supporting Information)),[2] it will behave mainly as the monoanion (LH^-) in a weakly basic solution (Eq. (S1)). In the absence of an enhancer agent, HRP(+3) in the resting state is firstly oxidized by H_2O_2 to Compound I(+5), (Eq. (S2)). Compound I(+5) is subsequently one-electron reduced with LH^- to yield a luminol radical ($LH\cdot$) and Complex II(4+), (Eq. (S3)). Further the reduction of Complex II(4+) with LH^- occurs to form another $LH\cdot$, resulting in the completion of a turnover of HRP (Eq. (S4)).[3, 4] The produced luminol radicals, possibly existing in equilibrium $LH\cdot \rightleftharpoons L^{-}\cdot + H^+$ (Fig. S1 (C) (Supporting Information)),[2] undergo the disproportionation to form luminol diazaquinone (Eq. (S5)); and subsequently, H_2O_2 existing in large excess amount reacts with luminol diazaquinone to give rise to the excited 3-aminophthalate (3-AP*) *via* the formation of the luminol dioxetane intermediate, followed by its decomposition (Eqs. (S6) and (S7)). Finally, 3-AP* emits photon when returning to the ground state (Eq. (S7)).[5] In the presence of an enhancer agent (referred to as SH), Compound I(+5) reacts with SH to produce enhancer radical (S·) and Compound II(+4) , which sequentially reacts with another SH to produce S· (Eqs. (S8) and (S9),[6, 7] followed by the formation of $LH\cdot$ *via* a reaction of S· with LH^- (Eq. (S10)). Following reaction steps are common to those free of an enhancer agent (Eqs. (S5), (S6), and (S7)).

Reference:

1. "*CRC Handbook of Chemistry and Physics*", Editor-in-Chief, J. R. Rumble, Jr., CRC Press, Boca Raton, **2019**, 5-104.
2. J. Lind, G. Merényi, and T. E. Eriksen, *J. Am. Chem. Soc.*, **1983**, *105*, 7655.
3. P. M. Prichard and M. J. Cormier, *Biochem. Biophys. Res. Commun.*, **1968**, *31*, 131.
4. M. J. Cormier and P. M. Prichard, *J. Biol. Chem.*, **1968**, *243*, 4706.
5. K. -D. Gundermann and F. McCapra, "*Chemiluminescence in Organic Chemistry*", **1987**, Springer-Verlag, Berlin, Heidelberg, New York, London, Paris, Tokyo.
6. T. P. Whitehead, L. J. Kricka, T. J. Carter, and G. H. Thorpe, *Clin. Chem.*, **1979**, *25*, 1531.
7. G. H. G. Thorpe and L. J. Kricka, in "*Methods in Enzymology*", ed. M. A. DeLuca and W. D. McElroy, Vol. 133, **1986**, Academic Press, Orlando, 331.

HRP activity against $(NH_4)_2SO_4$ concentration

Experimental procedures for the evaluation of the HRP activity in the presence of $(NH_4)_2SO_4$:

The experiments were carried out as follows; OPD was dissolved in the Tris (0.10 M)-HCl buffer solution (pH8.5) (10.0 mM) before use. The HRP solution was prepared from the HRP stock solution and diluted with the Tris (0.10 M) buffer solution (pH8.5) containing 3.5 M $(NH_4)_2SO_4$. The reaction was initiated according to the following manner: 1.50 cm^3 of 100.0 mM H_2O_2 solution containing 1000ppm EDTA with various concentrations of $(NH_4)_2SO_4$ was mixed with 1.50 cm^3 of the mixture of the Tris (0.10 M) buffer solution (pH8.5) containing various concentrations of $(NH_4)_2SO_4$ (5 vol) and 0.75 M NaOH (1 vol) in a quartz cell. The constituents of the final mixture are identical to those used for the CL reaction except for luminol-free. Next, 40 μL of 10.0 mM OPD was added to the mixture. Finally, 20 μL of 1.0×10^{-7} M HRP was added to the mixture and mixed rapidly, followed by monitoring absorbance at 417 nm at a 5 s interval for 5 min. In this system, the increase in absorbance at 417 nm corresponds to the formation of 2,3-diaminophenazine (DAP) (Eq. (S11)).[1]

$$2OPD + 3H_2O_2 \xrightarrow{HRP} \rightarrow\rightarrow DAP + 6H_2O \qquad (S11)$$

Reference:

1. S. Fornera and P. Walde, *Anal. Biochem.*, **2010**, *407*, 293.

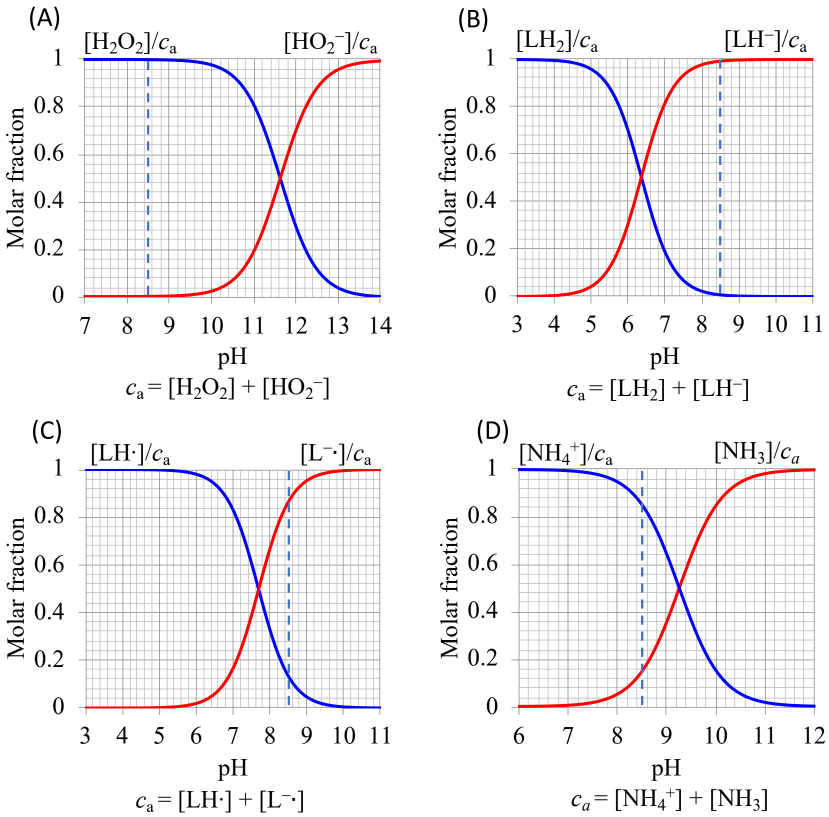

Fig. S1 Molar fraction of dissociated reactants as a function of pH.

(A) Hydrogen peroxide (H_2O_2), $pK_{a1} = 11.62$[1] (B) Luminol (LH_2), $pK_{a1} = 6.35$[2]

(C) Luminol radical ($LH\cdot$), $pK_a = 7.7$[2] (D) Ammonium ion (NH_4^+), $pK_a = 9.25$[1]

Vertical broken lines, pH of the reaction solution.

Calculation for molar fractions was carried out by using following equations:

$[HA]/c_a = [H^+]/([H^+] + K_a)$; $[A^-]/c_a = K_a/([H^+] + K_a)$

HA; H_2O_2, LH_2, or $LH\cdot$: A^-; HO_2^-, LH^-, or $L^-\cdot$

In the case of ammonium ion ($NH_4^+ \rightleftharpoons H^+ + NH_3$), the conjugate base concentration (= [A]) is used for calculation.

Reference:

1. "*CRC Handbook of Chemistry and Physics*", Editor-in-Chief, J. R. Rumble, Jr., CRC Press, Boca Raton, **2019**, 5-104.
2. J. Lind, G. Merényi, and T. E. Eriksen, *J. Am. Chem. Soc.*, **1983**, *105*, 7655.

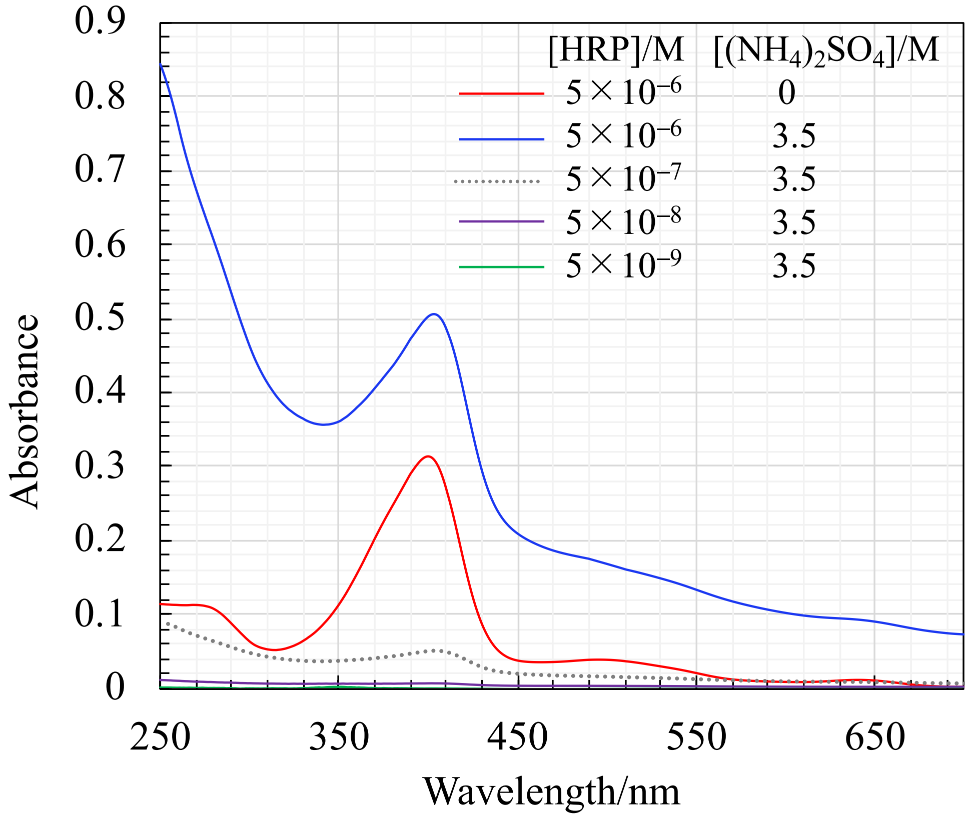

Fig. S2 Absorption spectra of various concentrations of HRP prepared with pH8.5 tris (0.10 M)-HCl buffer solution in the presence of 3.5 M $(NH_4)_2SO_4$ and in its absence. The resultant absorption spectra were subjected to the 21-point smoothing using JASCO Spectra Manager ver. 2.

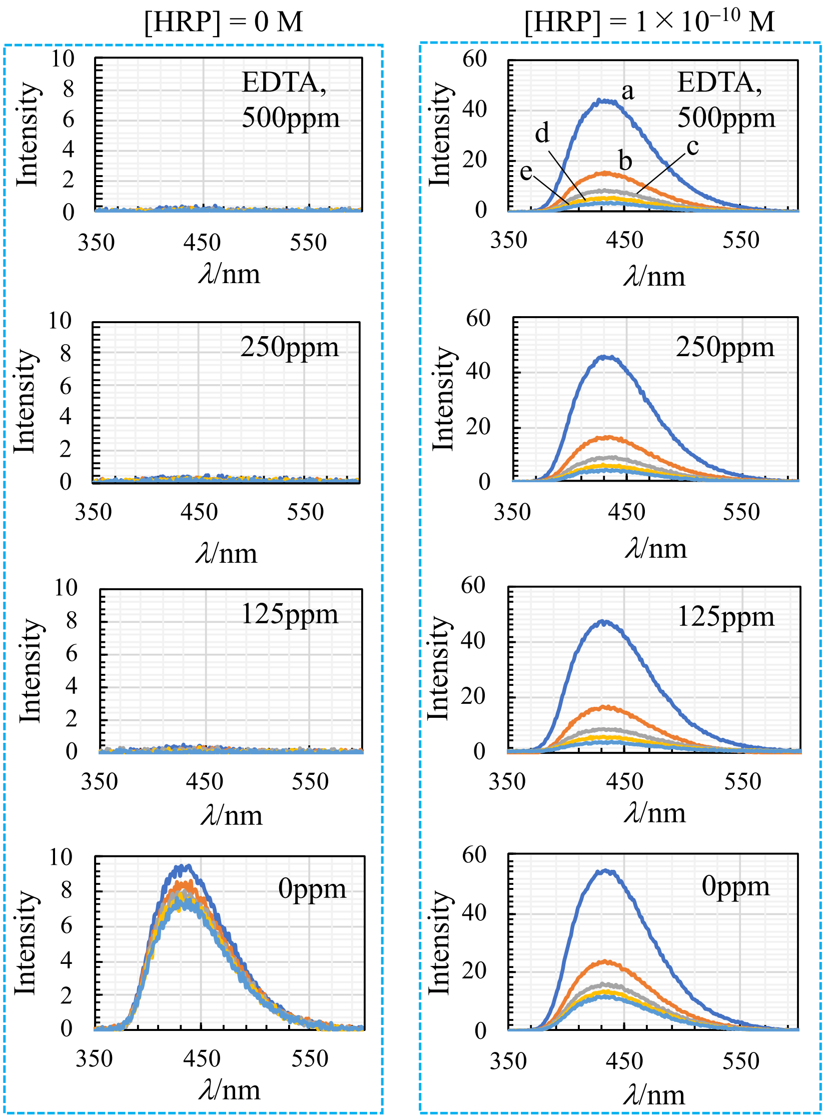

Fig. S3 Removal of background CL from luminol - H_2O_2 - HRP reaction in the presence of 3.2 M $(NH_4)_2SO_4$ by the addition of EDTA. Spectral measurement was carried out in a manner similar to those used for Fig. 1: a (blue), 0.166; b (orange), 1.166; c (gray), 2.166, d (yellow), 3.166; and e (light green), 4.166 min after the initiation of the reaction. Concentration of EDTA (ppm) in the reaction mixture is designated on each graph. Initial concentrations of H_2O_2 and luminol in the reaction mixture are 50.0 mM and 2.50 mM, respectively. HRP concentration in the reaction mixture; left dotted panel, 0 M; right dotted panel, 1.0×10^{-10} M. The ordinate scale for the CL reaction free of HRP is six times magnified as compared to that with 1.0×10^{-10} M HRP.

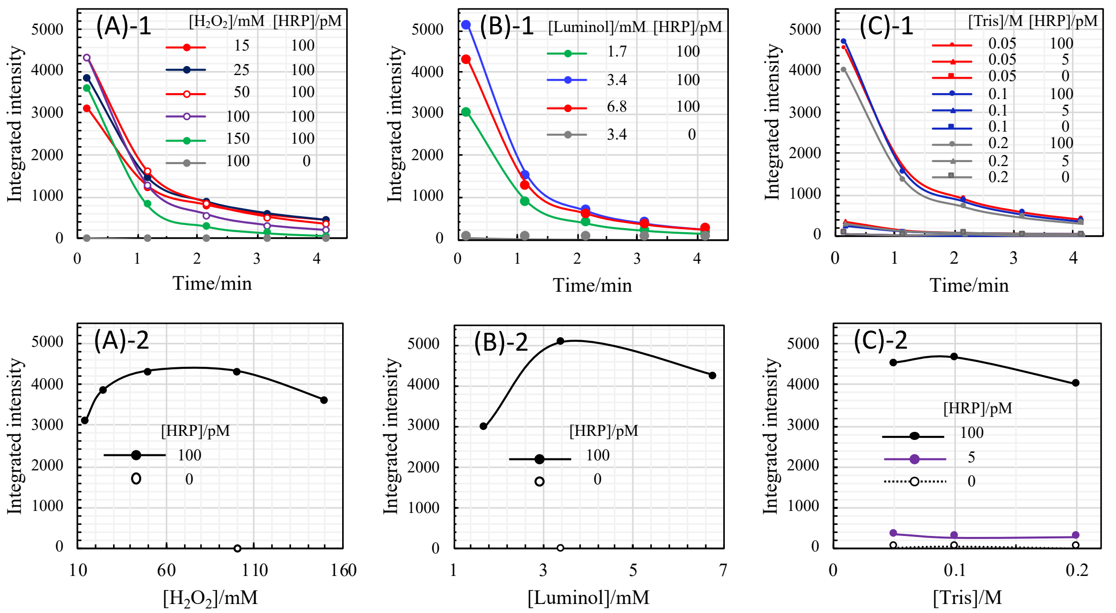

Fig. S4 Plots of integrated intensity against time after the initiation of the reaction at various concentrations of H_2O_2 (A), luminol (B), and Tris base (C). In all systems, concentrations of $(NH_4)_2SO_4$ and EDTA in the reaction mixture are 3.2 M and 500ppm. Concentrations in the reaction mixture: (A) luminol, 2.5 mM; Tris, 0.10 M: (B) H_2O_2, 50 mM; Tris, 0.10 M: and (C) H_2O_2, 50 mM; luminol, 2.5 mM. The chemiluminescence reaction was carried out in a manner similar to those used in Fig. 1. (A)-1, (B)-1, and (C)-1, CL time-courses; (A)-2, (B)-2, and (C)-2, plots of the integrated intensities of the spectrum recorded at 0.166 min against the concentration of each reagent. Open circles in (A)-2, (B)-2, and (C)-2, integrated intensity in the absence of HRP.

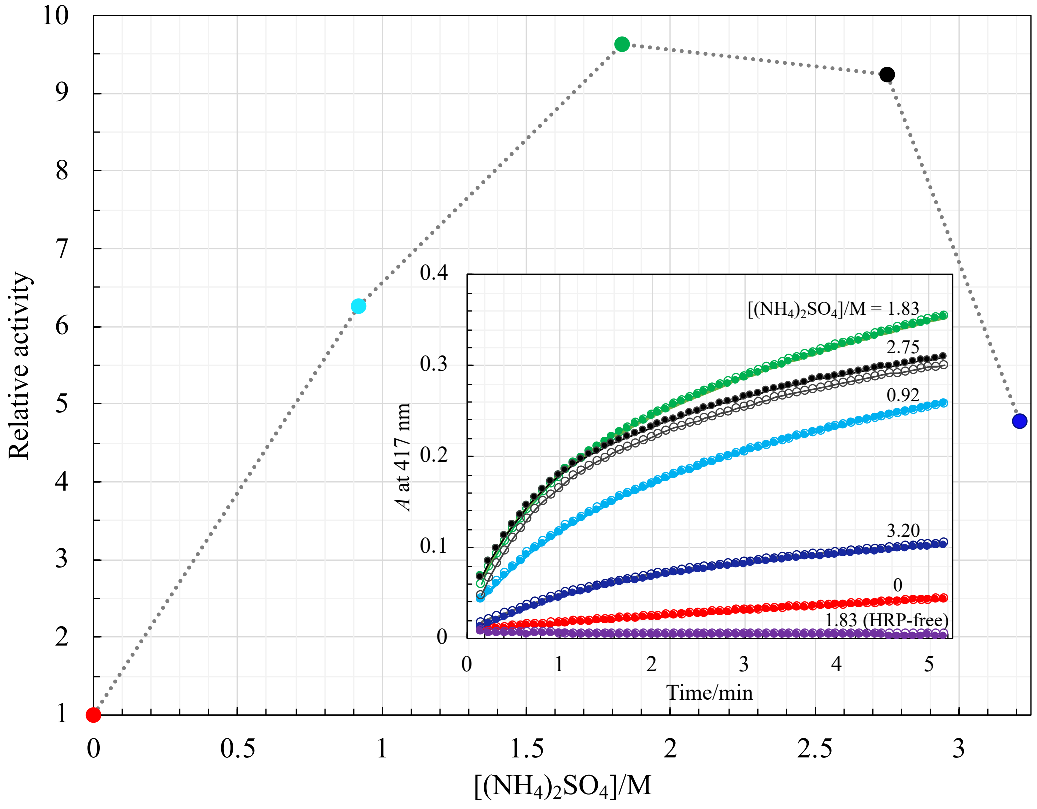

Fig. S5 Plots of relative activity of HRP against $(NH_4)_2SO_4$ concentration. Inset shows the time courses of absorbance at 417 nm at various concentrations of $(NH_4)_2SO_4$. Initial concentrations in the reaction mixture, [HRP] = 0.56 nM, $[H_2O_2]$ = 49 mM, [OPD] = 0.13 mM. Activity measurement was carried out twice in each system. $[(NH_4)_2SO_4]$/M in the reaction mixture; ●, 0; ●, 0.92 ; ●, 1.83; ●, 2.75; and ●, 3.20. ● (Inset), the composition of the reaction solution is identical to that used in the reaction of "●, 1.83" except for HRP-free.

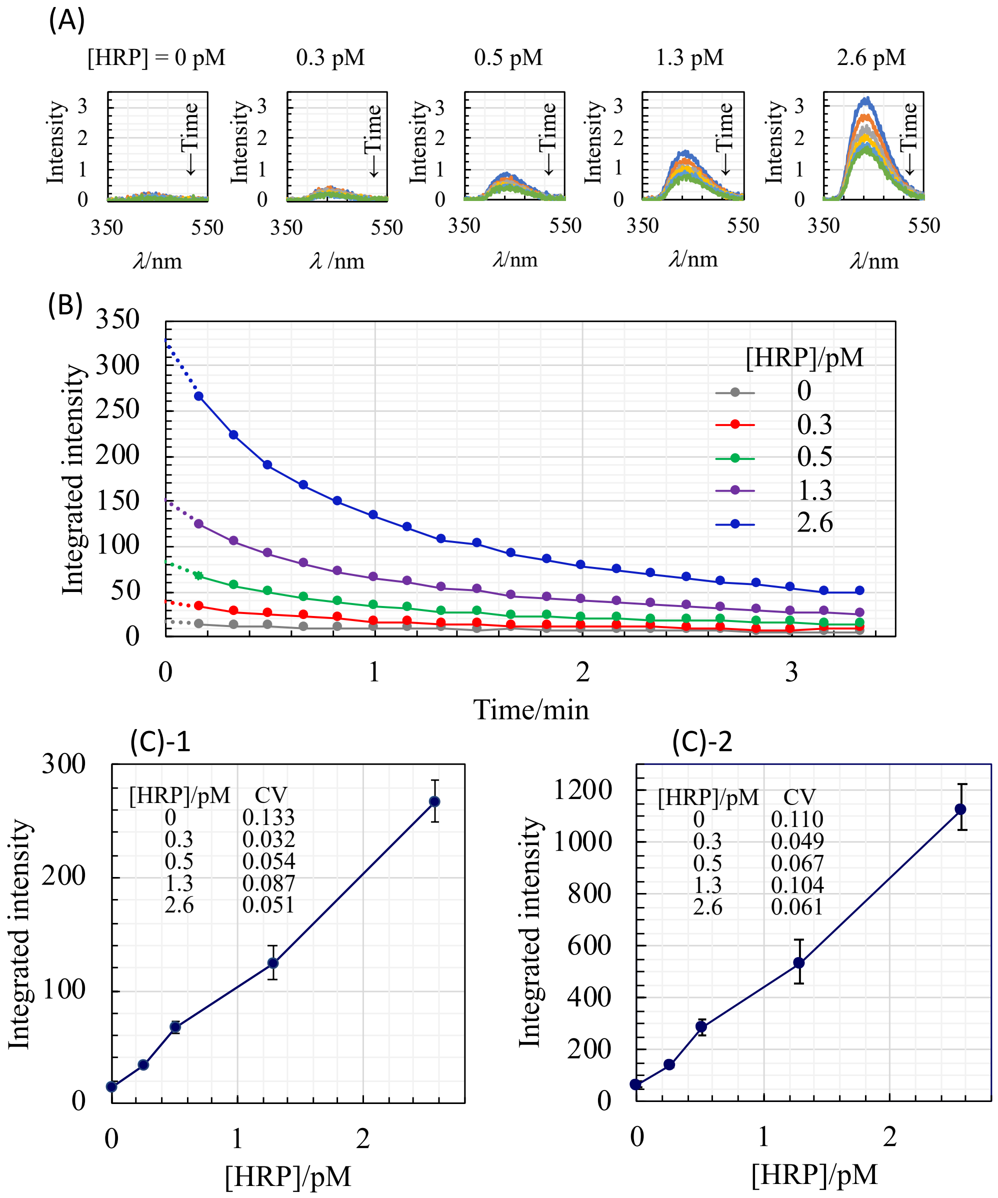

Fig. S6 CL output against HRP concentrations at sub-pM and pM levels in the presence of 3.2 M $(NH_4)_2SO_4$. (A) Changes in CL spectra (from 1st to 6th) as a function of time after the initiation of the reaction; CL spectra are obtained in a manner similar to that in Fig. 3. (B) Time-courses of CL outputs. All time-courses are extrapolated (broken line) to the time t = 0 according to the manner similar to that in Fig. 3. (C) Plots of CL intensities against the HRP concentration in the reaction mixture; (C)-1, the area of the first CL spectrum; and (C)-2, the total area of six spectra (from 1st to 6th). Coefficient of variation (CV = standard deviation/average intensity at each HRP concentration, n = 5) is designated in (C)-1 and (C)-2.
